# Supplementary material for: ALS-related FUS mutations alter axon growth in motoneurons and affect HuD/ELAVL4 and FMRP activity
Source: Commun Biol. 2021 Sep 1;4:1025. doi: 10.1038/s42003-021-02538-8 (PMC8410767; doi:10.1038/s42003-021-02538-8)
Supplement: Supplementary file 2 — Supplementary Information [file 42003_2021_2538_MOESM2_ESM.pdf]

SUPPLEMENTARY MATERIAL

ALS-related FUS mutations alter axon growth in motoneurons and affect HuD/ELAVL4 and FMRP activity

Maria Giovanna Garone[1], Nicol Birsa[2,3], Maria Rosito[4], Federico Salaris[1,4], Michela Mochi[1], Valeria de Turris[4], Remya R. Nair[5], Thomas J. Cunningham[5], Elizabeth M. C. Fisher[2], Mariangela Morlando[6], Pietro Fratta[2] and Alessandro Rosa[1,4,6,*]

1. Department of Biology and Biotechnology Charles Darwin, Sapienza University of Rome, P.le A. Moro 5, 00185 Rome, Italy
2. UCL Queen Square Institute of Neurology, University College London, London, WC1N 3BG, UK
3. UK Dementia Research Institute, University College London, London, WC1E 6BT, UK
4. Center for Life Nano- & Neuro-Science, Fondazione Istituto Italiano di Tecnologia (IIT), 00161 Rome, Italy
5. MRC Harwell Institute, Didcot, OX11 0RD, UK
6. Department of Pharmaceutical Sciences, "Department of Excellence 2018-2022", University of Perugia, Perugia, Italy.
7. Laboratory Affiliated to Istituto Pasteur Italia-Fondazione Cenci Bolognetti, Department of Biology and Biotechnology Charles Darwin, Sapienza University of Rome, Viale Regina Elena 291, 00161 Rome, Italy

* Corresponding author: alessandro.rosa@uniroma1.it; Tel: +39-0649255218

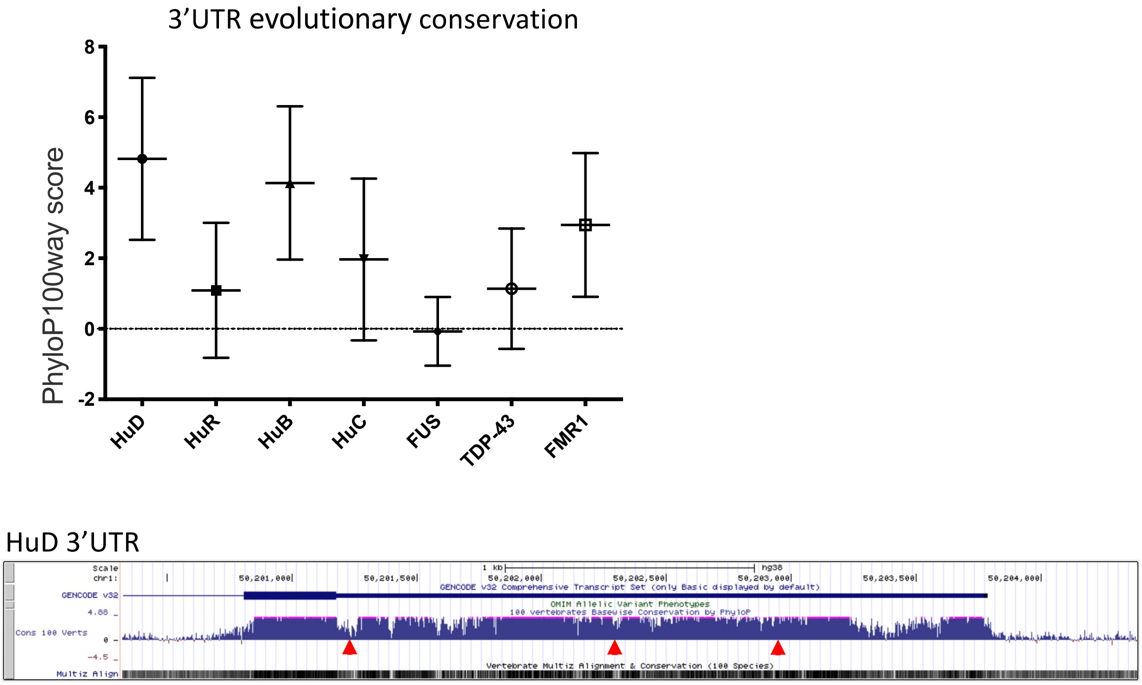

Supplementary Figure 1. Conservation of HuD/ELAVL4 3’UTR

Top: graph showing the mean and standard deviation values of the phyloP100way score relating to the 3’UTR sequences of indicated RBP genes (http://genome.ucsc.edu; UCSC Genome Browser assembly ID: hg38). Bottom: screenshot of the UCSC genome browser (http://genome.ucsc.edu; UCSC Genome Browser assembly ID: hg38) showing the sequence conservation of the HuD 3’UTR. Binding sites of miR-375 are indicated by red arrowheads.

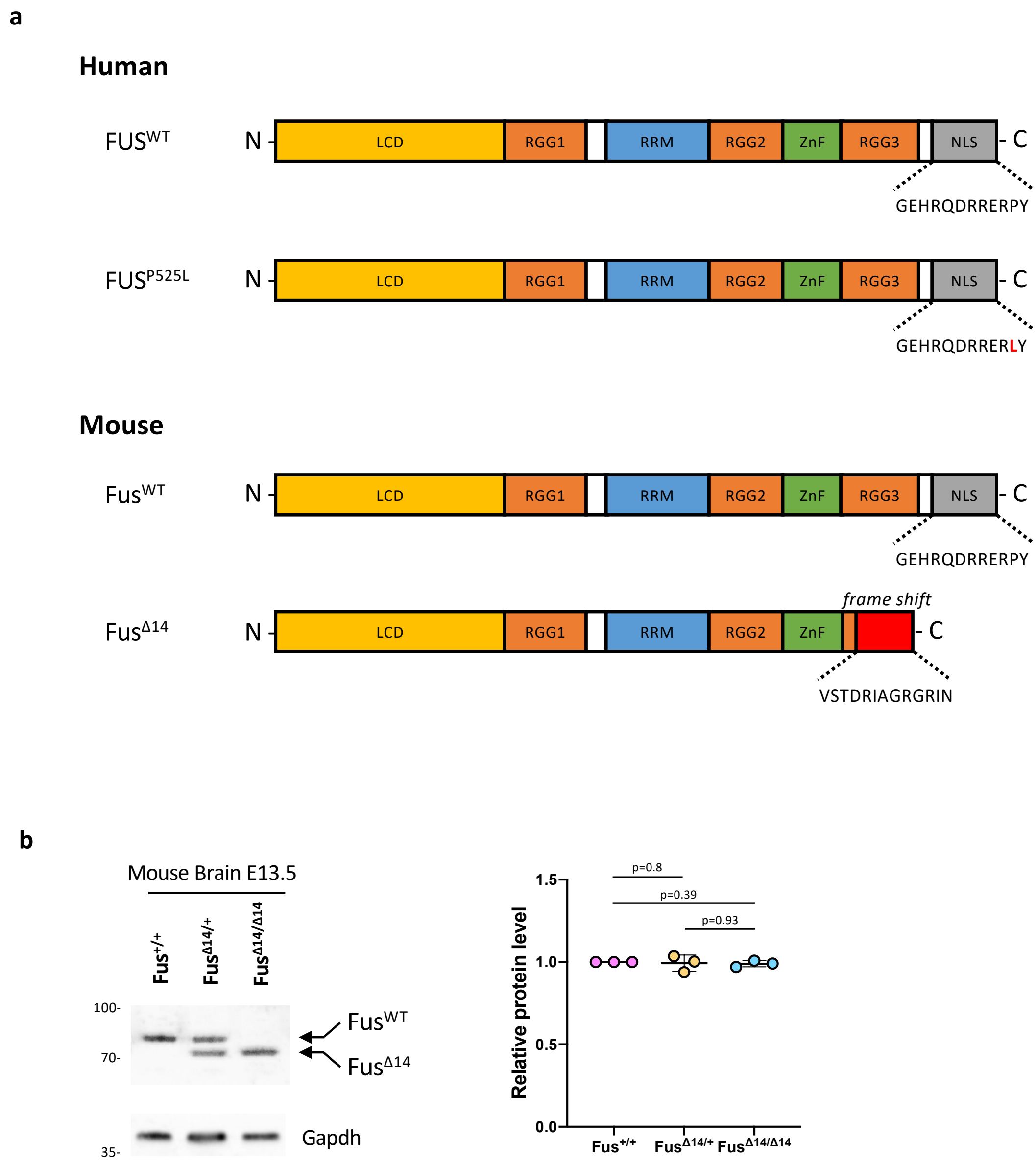

Supplementary Figure 2. FUS mutants used in this study

(a) Schematic representation of human and mouse FUS mutants used in this study. (b) Western blot analysis and quantification of wild-type and mutant Fus protein levels in the brain of mouse models used in this study at E13.5. Gapdh signal was used for normalization. Protein levels are relative to $Fus^{+/+}$ conditions. The molecular weight (kDa) is indicated on the left. The graph shows the average from 3 independent biological replicates, error bars indicate the standard deviation (ordinary one-way ANOVA, multiple comparisons).

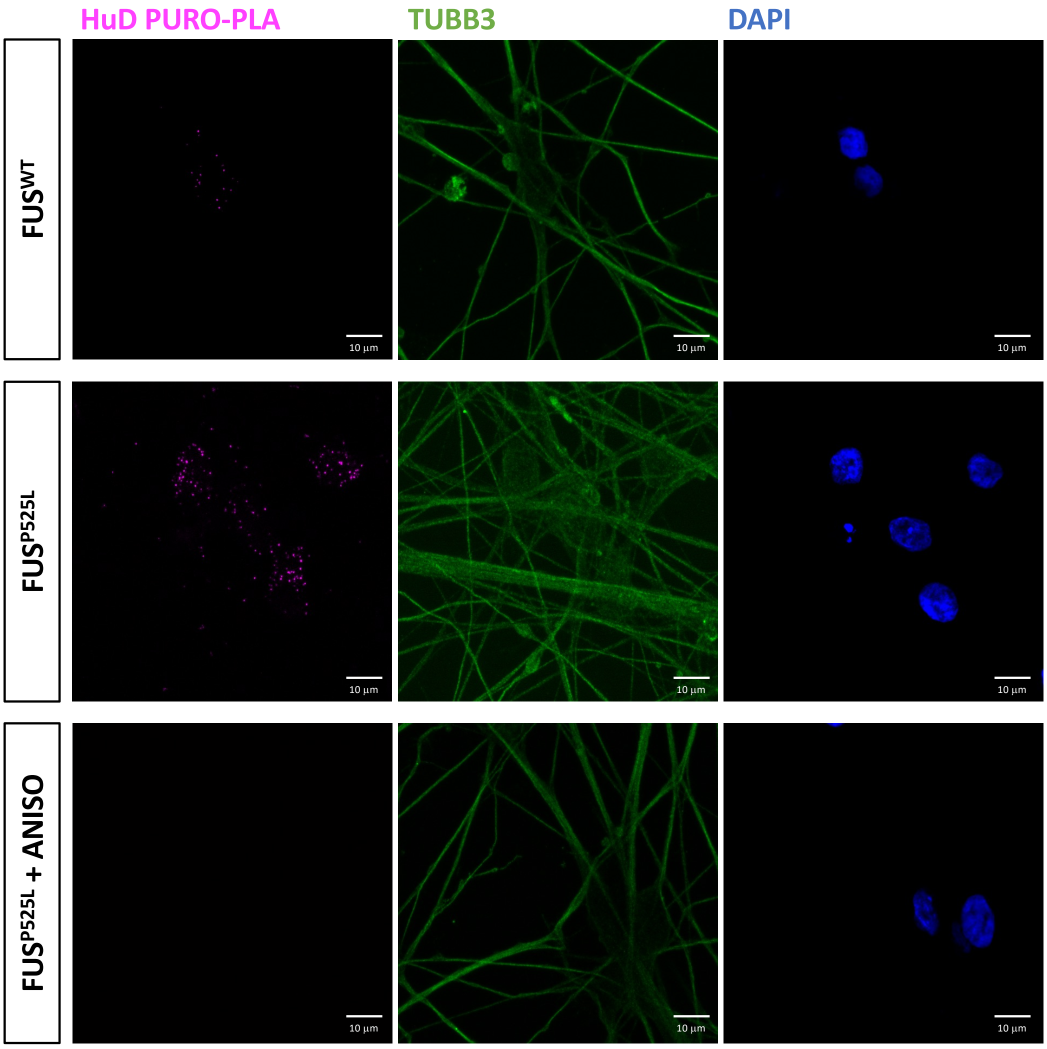

Supplementary Figure 3. HuD PURO-PLA and TUBB3 immunostaining analysis

Single panels of the PURO-PLA (HuD, magenta) and immunostaining (TUBB3, green) analysis in FUS^{WT} and FUS^{P525L} hiPSC-derived spinal MNs, shown in Figure 2. DAPI (blue) was used for nuclear staining. Cells treated with the eukaryotic protein synthesis inhibitor anisomycin (FUS^{P525L} + ANISO), used as a PURO-PLA control, are also shown. Scale bar: 10 µm.

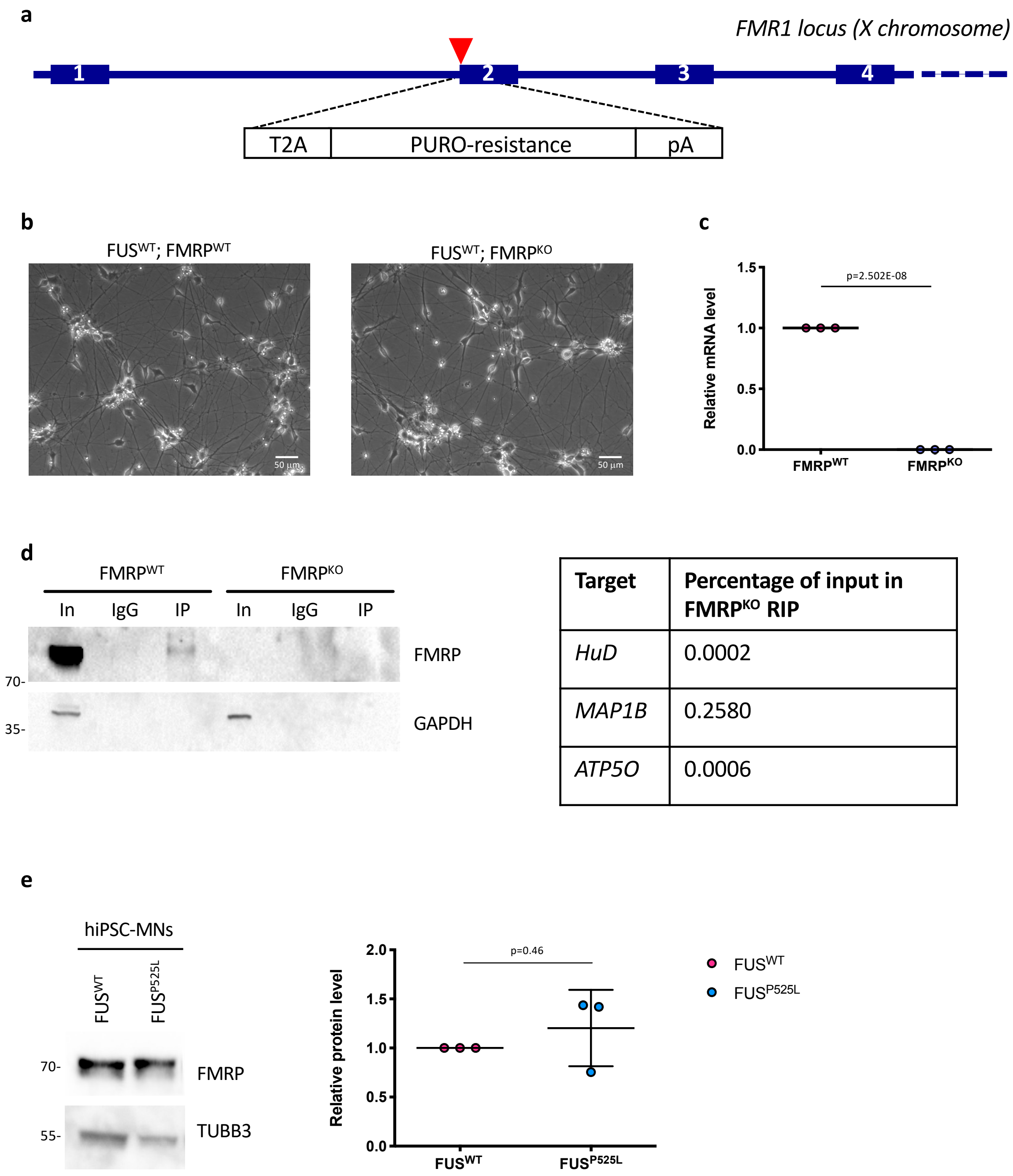

Target	Percentage of input in FMRP^{KO} RIP
HuD	0.0002
MAP1B	0.2580
ATP5O	0.0006

Supplementary Figure 4. FMR1 knock-out hiPSCs

(a) Schematic representation of the FMR1 locus and the strategy for CRISPR/Cas9 knockout. The red triangle indicates the target site of the CRISPR guides. The cassette encoding for the self-cleavage peptide T2A, the puromycin resistance gene (PURO-resistance) and the BGH cleavage and polyadenylation site (pA), schematized in the figure, was integrated in the genome upon homology

directed repair, interrupting the FMR1 coding sequence. Selection with puromycin was followed by clonal expansion and characterization of the FMR1 knock-out line, as described[1]. (b) Phase contrast images of hiPSC-derived MNs obtained from the parental and the FMRP knock-out targeted lines. Scale bar: 50 µm. (c) Analysis of the FMR1 mRNA levels by real time qRT-PCR in $FMRP^{WT}$ and $FMRP^{KO}$ hiPSC-derived MNs. The graph shows the average from 3 independent differentiation experiments, error bars indicate the standard deviation (Student's t-test, paired, two tails). (d) Left: western blot analysis of the FMRP RIP assay, as in Figure 2a, performed on hiPSC-derived MNs obtained from the parental and the FMRP knock-out targeted lines. The molecular weight (kDa) is indicated on the left. Right: table showing the results of the FMRP RIP analysis of MNs derived from the FMR1 knock-out line, used as a negative control of the experiments shown in Figure 2a,b. (e) FMRP protein levels analysis by western blot in FUS^{WT} and FUS^{P525L} hiPSC-derived spinal MNs. The molecular weight (kDa) is indicated on the left. The graph shows the average from 3 independent differentiation experiments, error bars indicate the standard deviation (Student's t-test, paired, two tails). TUBB3 signal was used for normalization. Protein levels are relative to FUS^{WT} conditions.

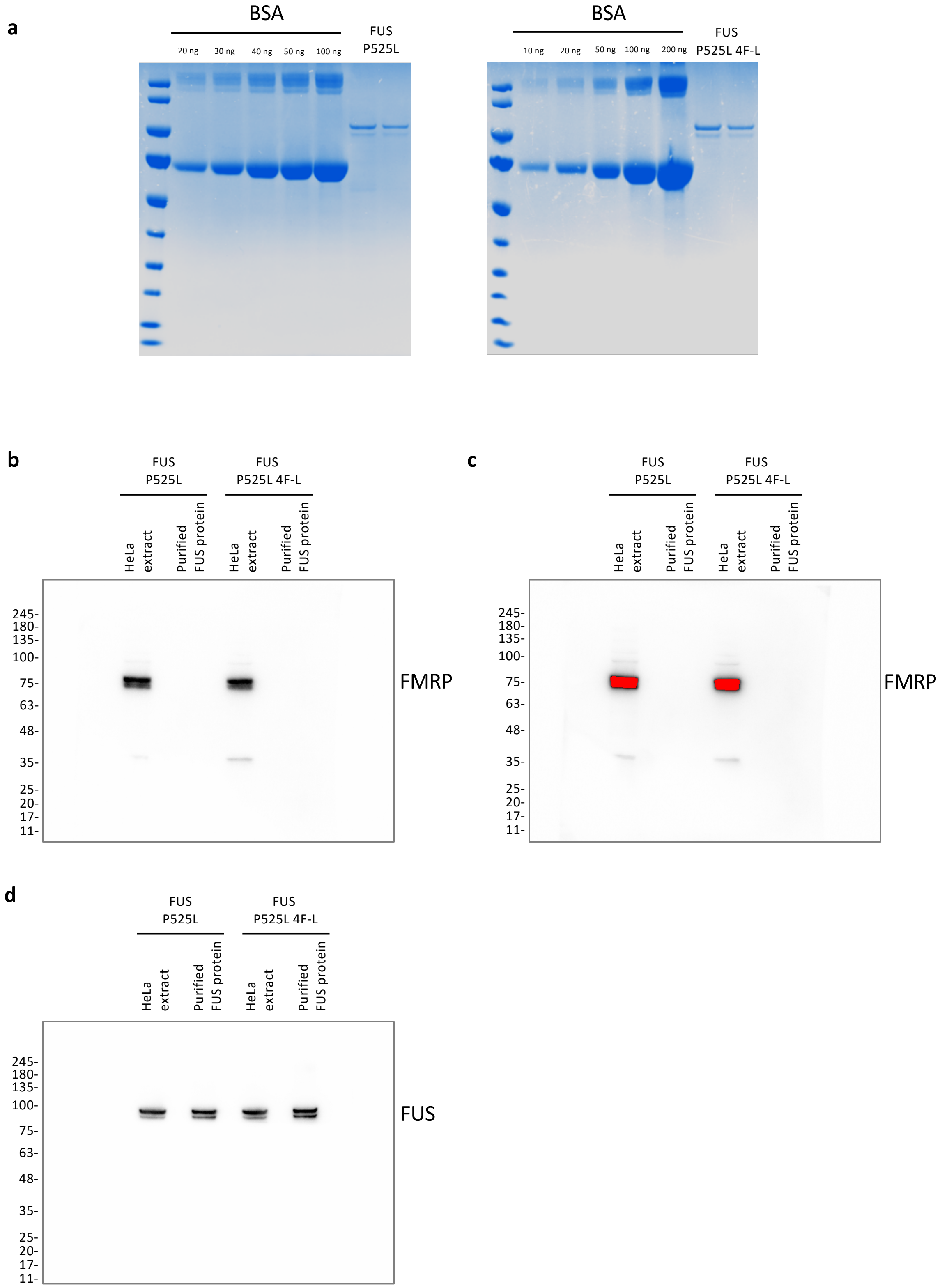

Supplementary Figure 5. Purification of the recombinant FUS protein from HeLa extracts

(a) Recombinant flag-tagged FUS proteins (left: P525L; right: P525L 4F-L) were purified from HeLa extracts and run on an electrophoretic gel together BSA at known concentrations for quantification.

Coomassie blue staining of the gels confirmed effective purification of the recombinant proteins. (b-d) Western blot analysis of FMRP (b,c) and recombinant flag-tagged FUS (d; using an anti-flag antibody) in HeLa extracts (8 μg) and purified recombinant flag-tagged FUS protein samples (3 ng). Panel (c) shows the same filter as in (b) at higher exposure time, beyond saturation of the FMRP signal in HeLa extract samples. The molecular weight (kDa) is indicated on the left. This analysis confirms no detectable contamination of FMRP in purified recombinant flag-tagged FUS protein samples from HeLa extracts.

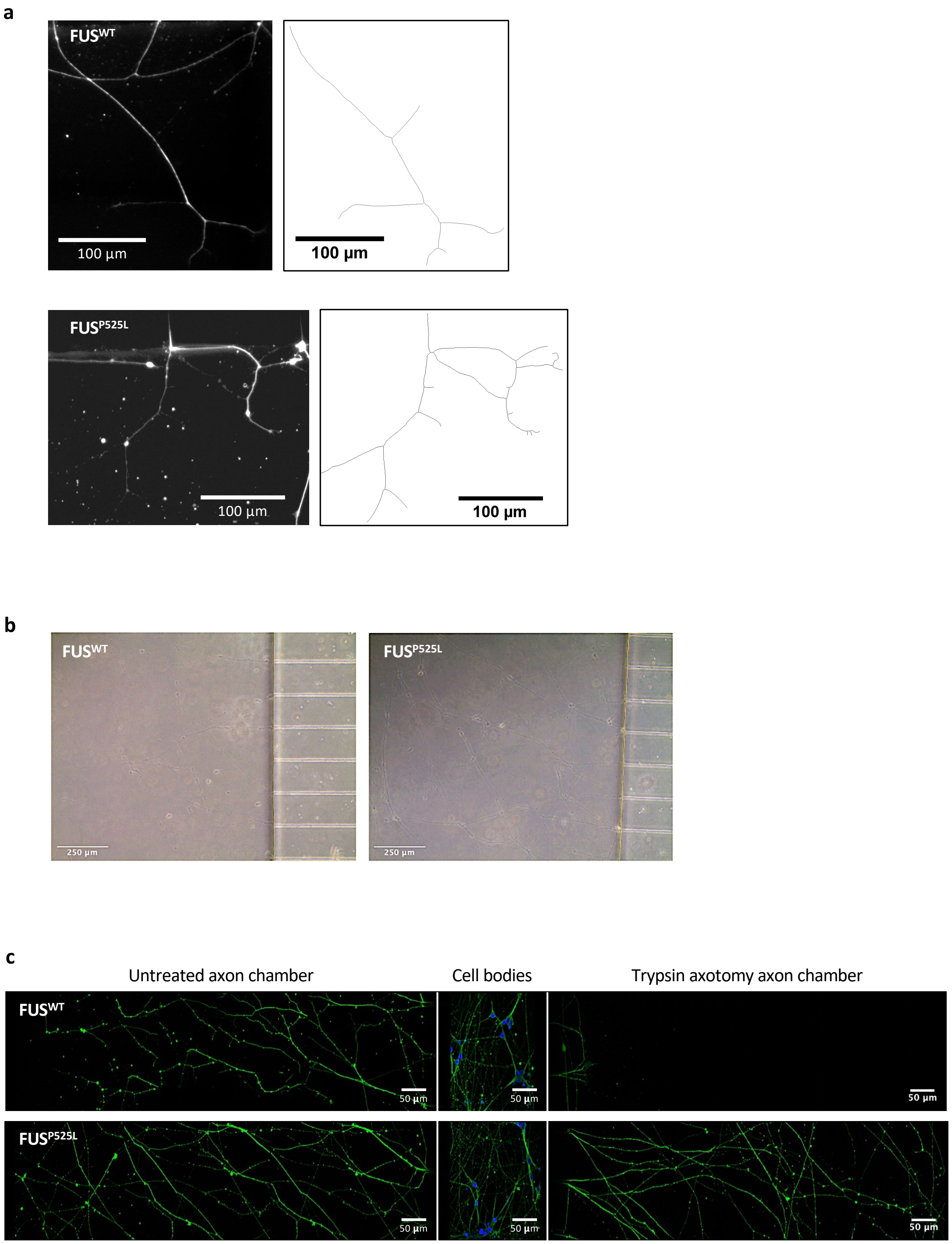

Supplementary Figure 6. Axon branching analysis and axotomy and recovery assays

(a) Immunofluorescence images (TUBB3 in white; left) and corresponding images generated with the Skeleton plugin of ImageJ (right), showing axons of FUS^{WT} and FUS^{P525L} hiPSC-derived MNs in the axon chamber of compartmentalized chips. Scale bar: 100 µm. (b) Brightfield images of the axons,

before trypsin axotomy, of FUS^{WT} and FUS^{P525L} hiPSC-derived MNs in the axon chamber of compartmentalized chips. Scale bar: 250 μm. (c) Immunostaining of TUBB3 (green) in FUS^{WT} and FUS^{P525L} hiPSC-derived spinal MNs cultured in compartmentalized chips and allowed to recover for 30 hours after the trypsin treatment to induce axotomy. DAPI (blue) was used for nuclear staining. The figure shows the trypsin treated axon chamber on the right, the untreated axon chamber, used as control, on the left and the cell body chamber in the middle. Scale bar: 50 μm.

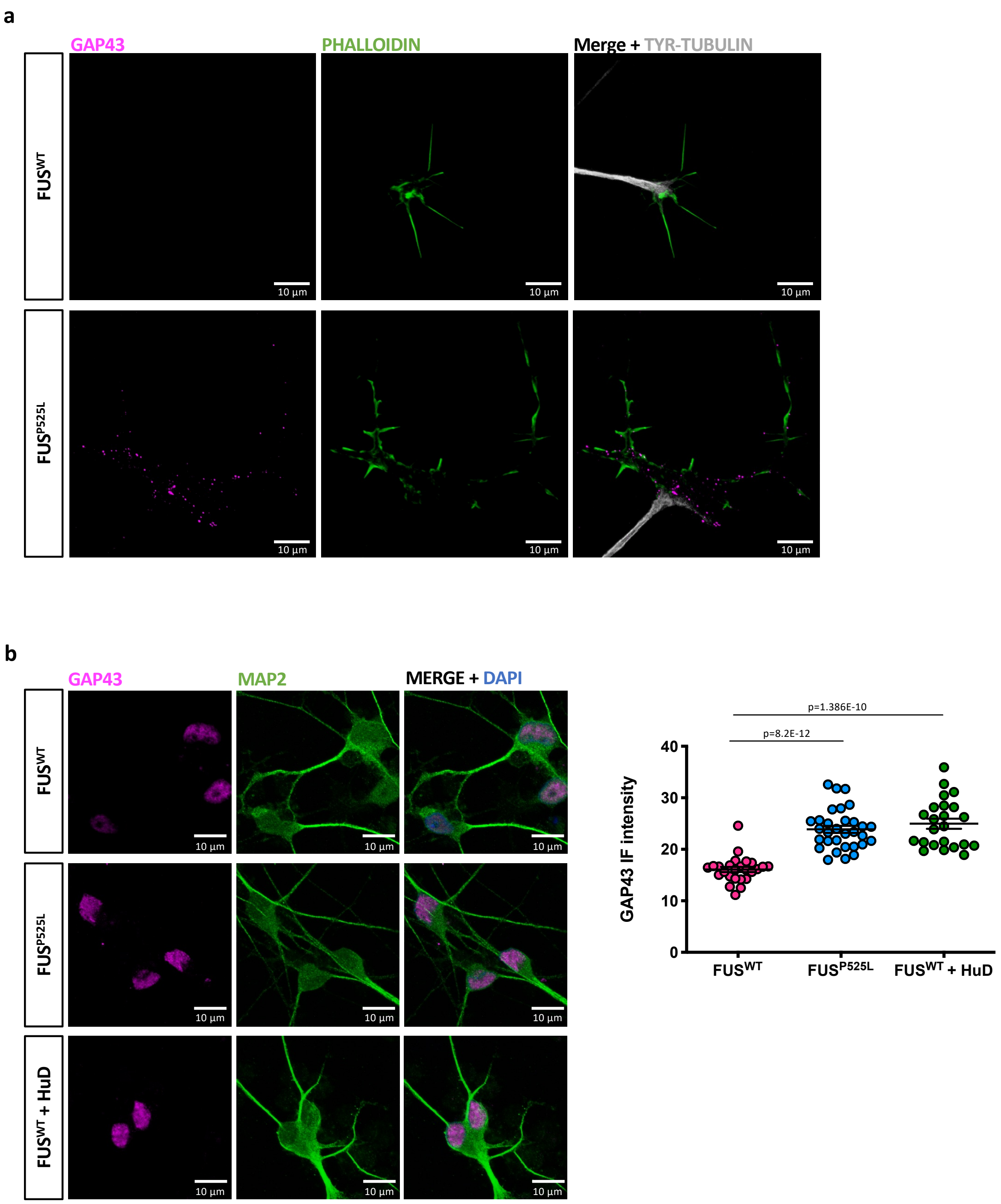

Supplementary Figure 7. Immunostaining analysis of GAP43 in the soma

(a) Immunostaining analysis in FUS^{WT} and FUS^{P525L} hiPSC-derived spinal MNs growth cones (individual panels related to Figure 7d). GAP43 signal is magenta; PHALLOIDIN signal (marking growth cones) is green. Images on the right show the merge of GAP43, PHALLOIDIN and TYR-TUBULIN (tyrosinated alpha-tubulin; marking axons) (white). Scale bar: 10 µm. (b) Immunostaining analysis in FUS^{WT}, FUS^{P525L} and FUS^{WT} overexpressing HuD under the SYN1 promoter

(FUS^{WT}+HuD) hiPSC-derived spinal MNs. GAP43 signal is magenta; MAP2 signal is green. DAPI (blue) was used for nuclear staining. The graph shows the NRN1 signal intensity from 3 independent differentiation experiments, error bars indicate the standard error of the mean (Student's t test; unpaired; two tails). Scale bar: 10 µm.

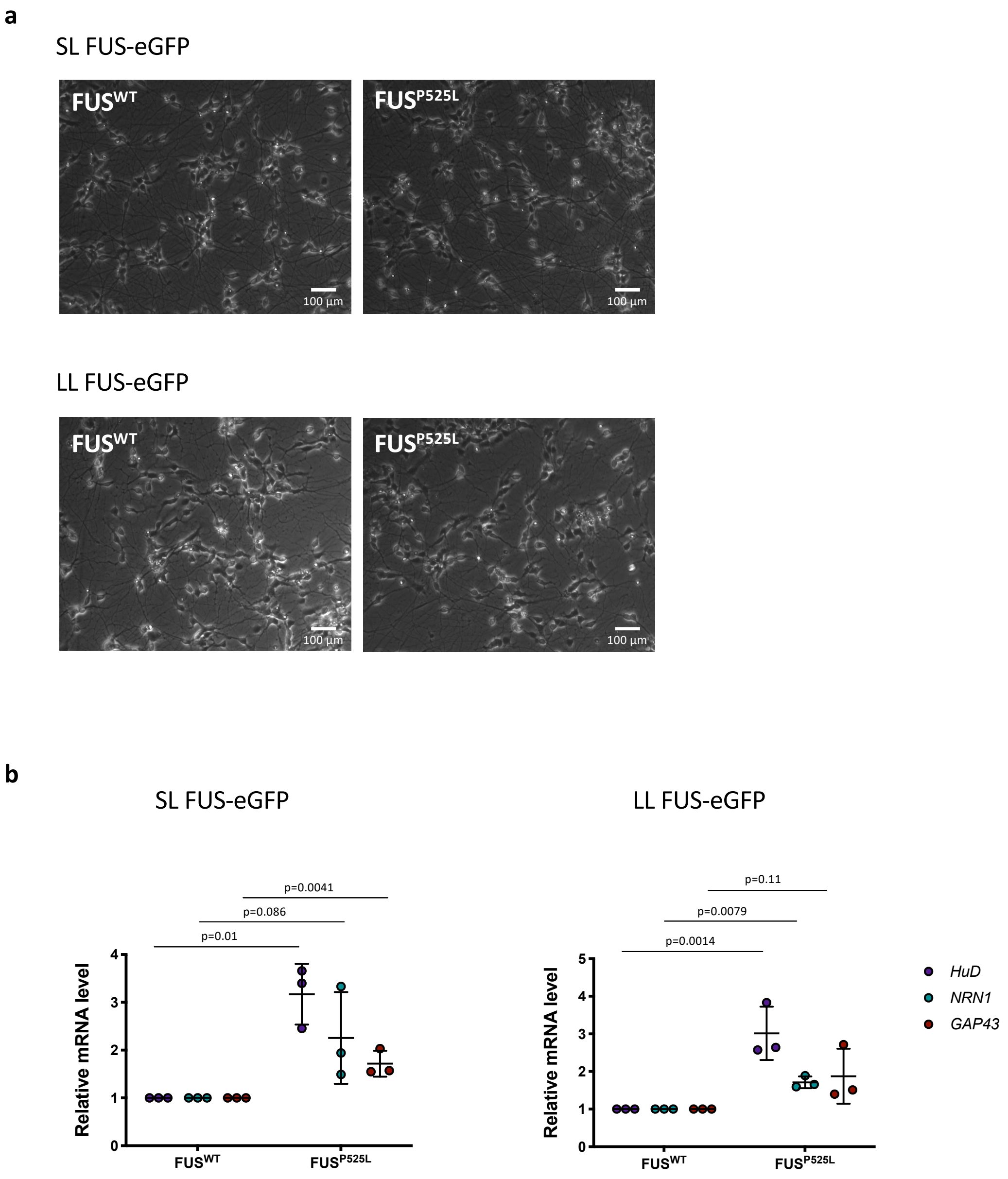

Supplementary Figure 8. HuD, NRN1 and GAP43 mRNA levels in MNs from independent FUS mutant hiPSC lines

(a) Phase contrast images of MNs obtained by differentiation of KOLF hiPSCs WT 2 and P525L16 (LL FUS-eGFP) and T12.9 hiPSCs WT15 and P525L17 (SL FUS-eGFP), a kind gift of J. Sterneckert[2]. Scale bars: 50 µm. (b) Analysis of the mRNA levels of the indicated genes by real time qRT-PCR in MNs shown in panel (a). The graph shows the average from 3 independent differentiation experiments, error bars indicate the standard deviation (Student's t-test; paired; two tails).

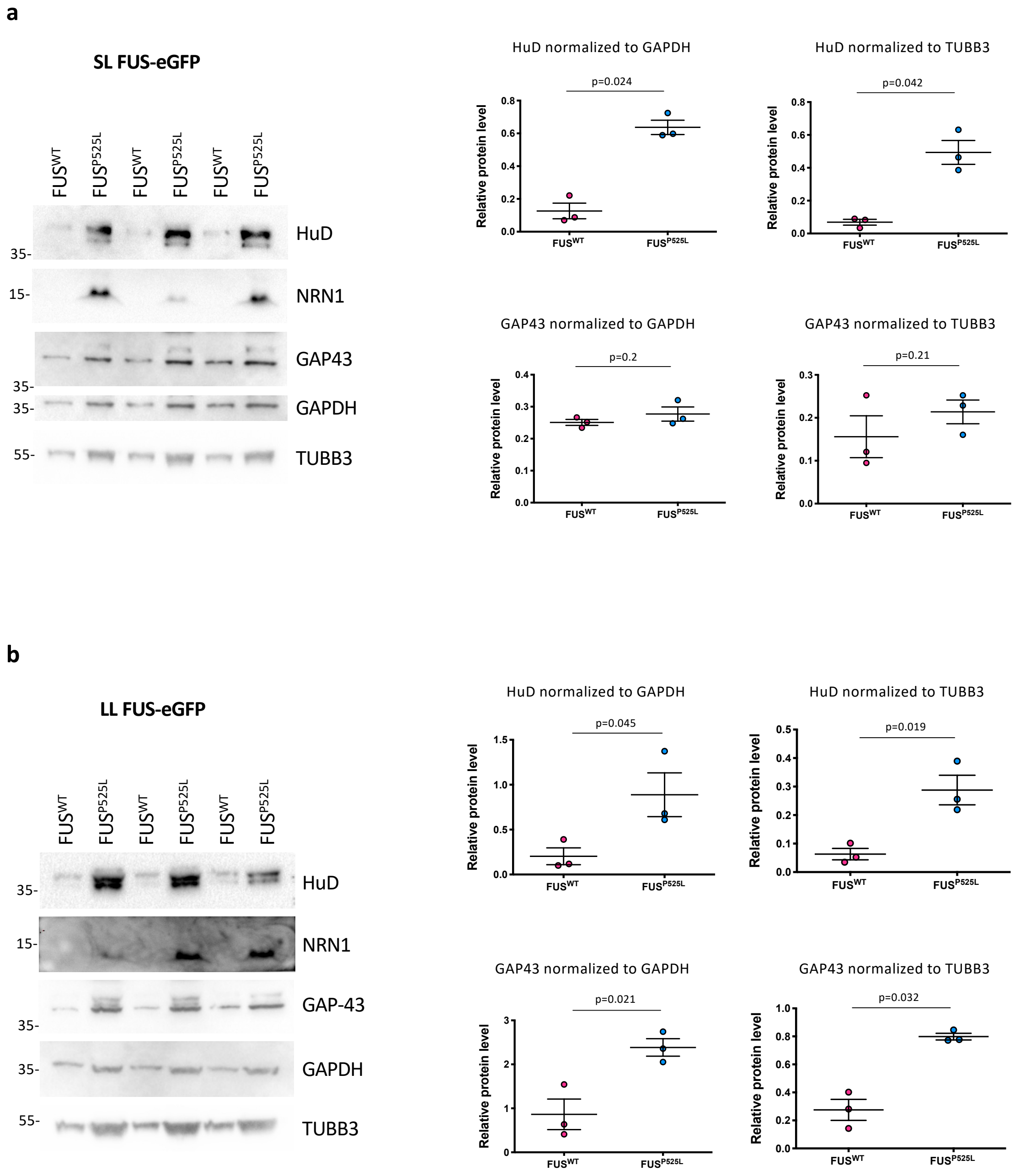

Supplementary Figure 9. HuD, NRN1 and GAP43 protein levels in MNs from independent FUS mutant hiPSC lines

(a,b) Analysis by western blot, of the levels of the indicated proteins in hiPSC-derived MNs shown in Supplementary Figure 8. Blots relating to 3 independent differentiation experiments are shown. The molecular weight (kDa) is indicated on the left. The graphs show the quantification of the western blot signals normalized to TUBB3 or GAPDH, as indicated, and protein levels are relative to FUS^{WT} conditions, error bars indicate the standard deviation (Student's t-test; paired; two tails).

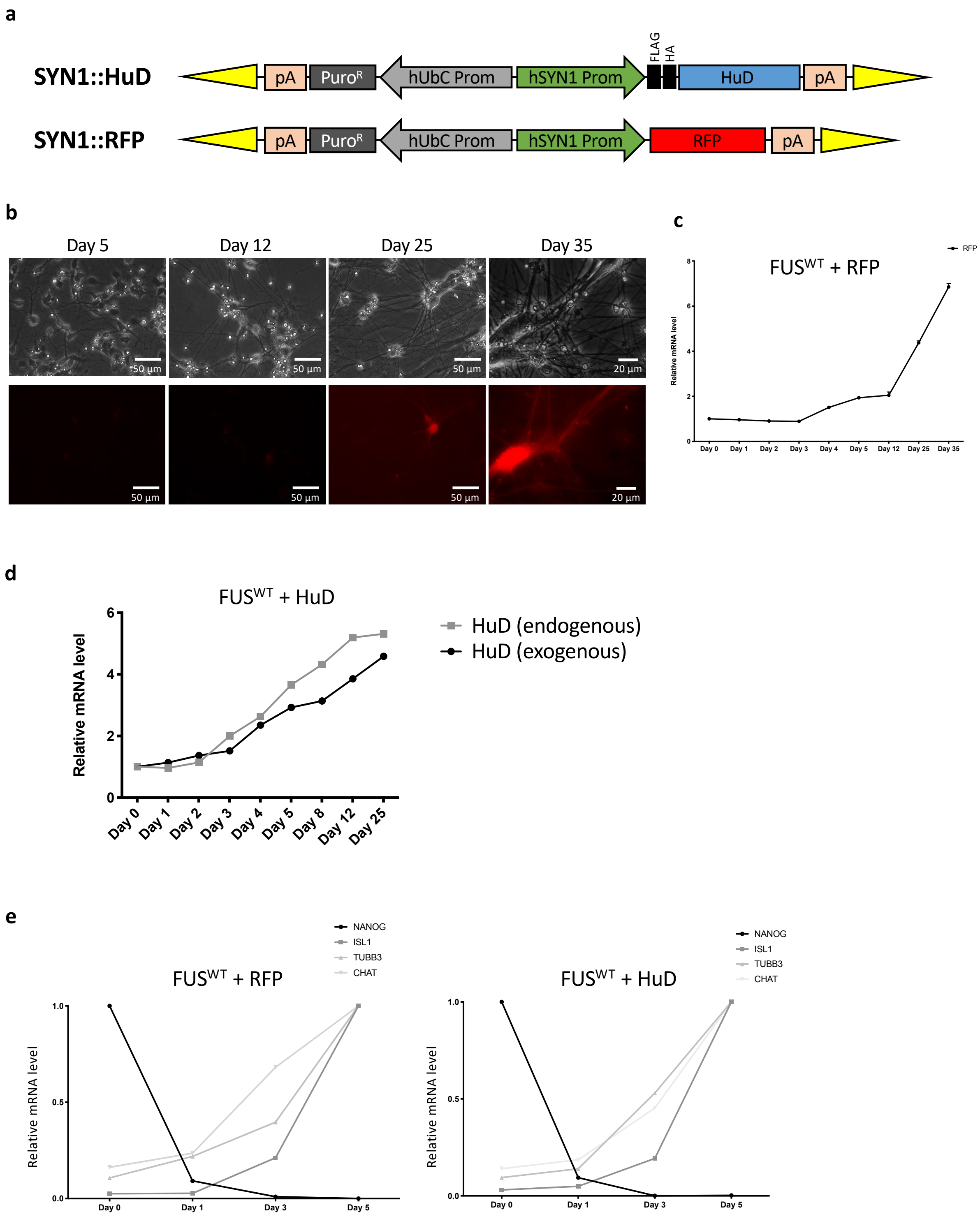

Supplementary Figure 10. Validation of the SYN1::HuD construct

(a) Schematic representation of the enhanced piggyBac (epB)-based vectors for the expression of HuD (top) or tagRFP (bottom) under the human synapsin 1 promoter (hSYN1 prom). Yellow triangles represent epB terminal repeats. pA: cleavage and polyadenylation site; Puro^R: puromycin resistance gene; hUbC Prom: constitutive promoter of the human Ubiquitin C gene. FLAG:

DYKDDDDK tag. HA: human influenza hemagglutinin molecule tag. (b) Phase contrast (top) and RFP fluorescence (bottom) images of hiPSCs stably transduced with the tagRFP-encoding epB vector shown in panel A and induced to differentiate to MNs. Time points of differentiation are shown on top of the panels. Scale bars: 20 μm. (c) Real time qRT-PCR analysis of tagRFP expression in differentiating cells as in (b). (d) Expression of exogenous and endogenous HuD mRNA detected by real time qRT-PCR with specific primers. (e) Real time qRT-PCR analysis of the indicated marker genes in differentiating hiPSCs, showing that exogenous HuD expression does not alter MN differentiation.

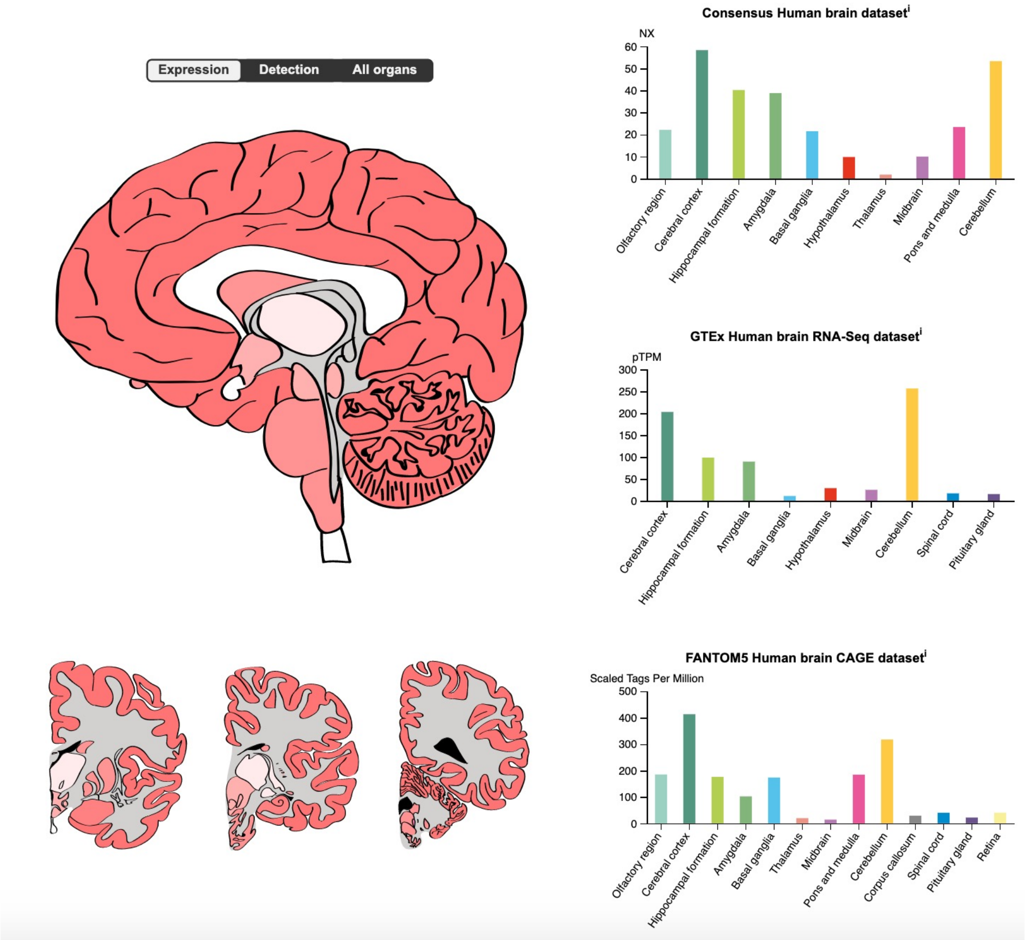

Supplementary Figure 11. Expression of NRN1 in human nervous system

Image and data available from v19.3.proteinatlas.org[3]. The Human Protein Atlas is available from http://www.proteinatlas.org and licensed under the Creative Commons Attribution-ShareAlike 3.0 International License.

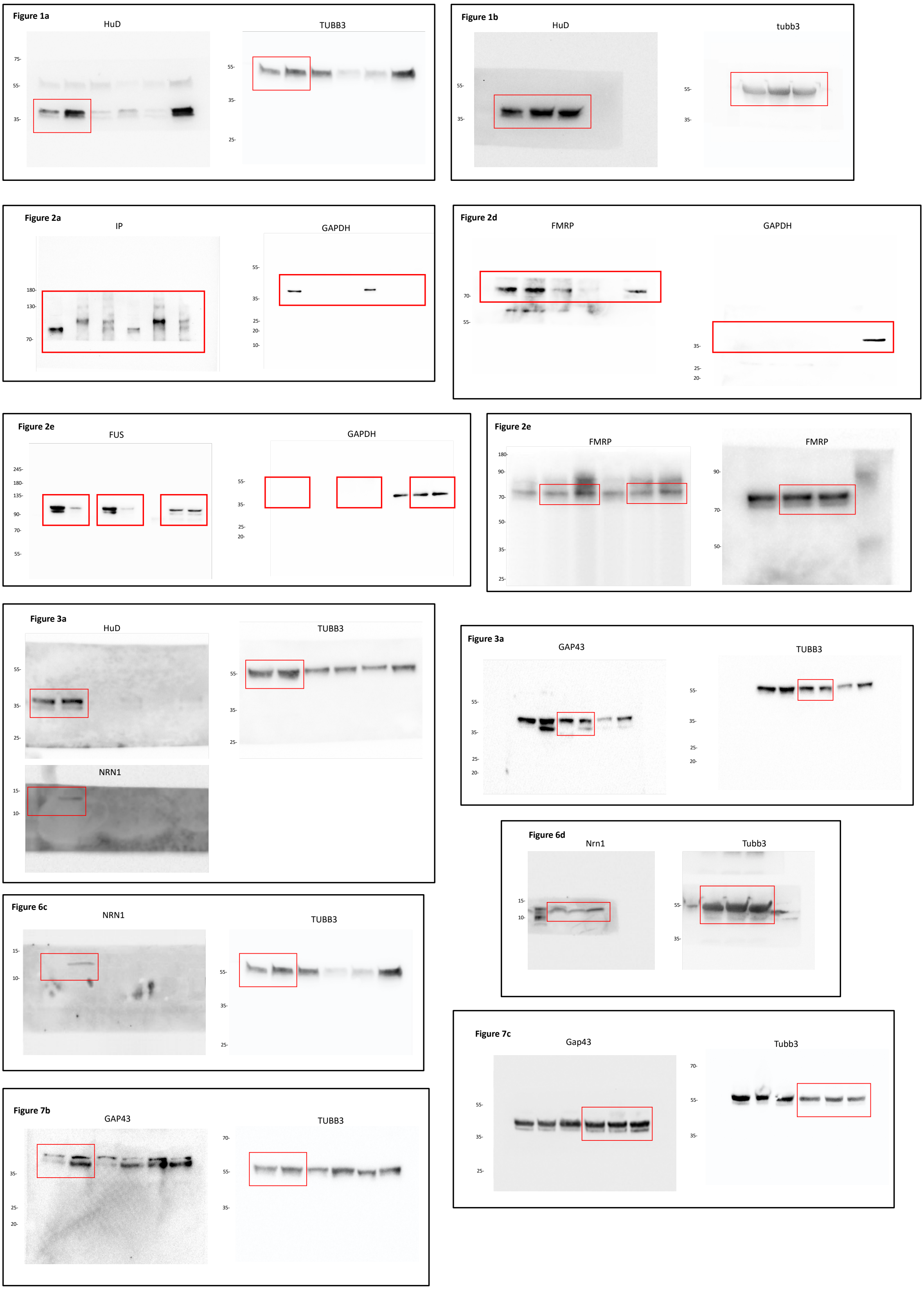

Supplementary Figure 12. Uncropped blots for main figures

Uncropped images for the western blots shown in the main figures of the paper. Red boxes indicate the cropped bands shown in the figures. The molecular weight (kDa) is indicated on the left.

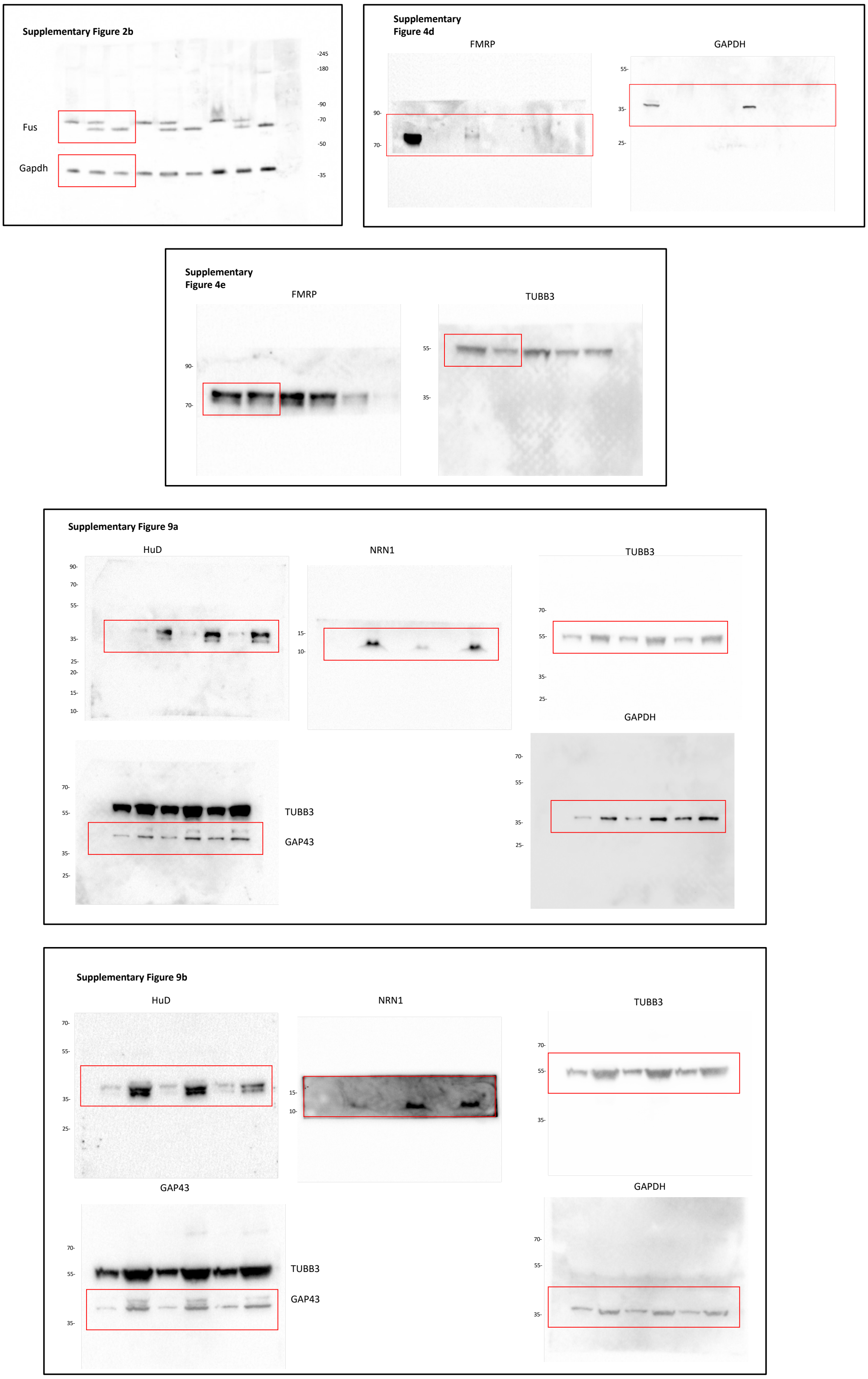

Supplementary Figure 13. Uncropped blots for supplementary figures

Uncropped images for the western blots shown in the supplementary figures of the paper. Red boxes indicate the cropped bands shown in the figures. The molecular weight (kDa) is indicated on the left.

MGI	Symbol	Name	Annotated Term	Evidence	Inferred From	Reference(s)
1099460	RBM3	RNA binding motif (RNP1, RRM) protein 3	positive regulation of translation	ISO	RGD:620145	J:155856
108177	DHX9	DEAH (Asp-Glu-Ala-His) box polypeptide 9	positive regulation of cytoplasmic translation	ISO	Q08211	J:164563
1100865	RBM4	RNA binding motif protein 4	positive regulation of translation	IDA		J:119742
1100851	ELAVL1	ELAV (embryonic lethal, abnormal vision)-like 1 (Hu antigen R)	positive regulation of translation	IDA		J:175944
107427	ELAVL4	ELAV like RNA binding protein 4	regulation of translation at synapse, modulating synaptic transmission	ISO	RGD:1560027	J:155856
95564	FMR1	FMRP translational regulator 1	negative regulation of cytoplasmic translation	IDA		J:231207
107914	TIA1	cytotoxic granule-associated RNA binding protein 1	negative regulation of translation	IMP	MGI:3037091	J:88770

Supplementary Table 1. Candidate regulators of HuD translation

The table shows RBPs predicted to bind HuD 3'UTR by *cat*RAPID[4], which are also known regulators of translation. Gene Ontology Evidence Code Abbreviations: ISO, inferred from sequence orthology; IDA, inferred from direct assay; IMP, inferred from mutant phenotype.

ATP5O FW	ACTCGGGTTTGACCTACAGC
ATP5O RV	GGTACTGAAGCATCGCACCT
HuD FW	CAACCCCAGCCAGAAGTCCA
HuD RV	AGCCTGAACCTCTGAGCCTG
NRN1 FW	GGCTTTTCGGACTGTTTGCTCA
NRN1 RV	ATCCTCCCAGTATGTGCACACG
SYN-Flag-HuD FW	ACTCAGCGCTGCCTCAGTCTG
SYN-Flag-HuD RV	ACCTGAGGCTCCATGGTGCTAAT
RFP FW	AACACTCGGCTGGGAGGCCAA
RFP RV	ACGTAGGTCTCTTTGTCGGCCT
NANOG FW	CCAAATTCTCCTGCCAGTGAC
NANOG RV	CACGTGGTTTCCAAACAAGAAA
TUJ1 FW	CCCGGAACCATGGACAGTGT
TUJ1 RV	TGACCCTTGGCCCAGTTGTT
CHAT FW	CTCAGCTACAAGGCCCTGCT
CHAT RV	ACCAGCGTGTCCTGGGTATG
ISL-1 FW	TACAAAGTTACCAGCCACC
ISL-1 RV	GGAAGTTGAGAGGACATTGA
GAP-43 FW	GAGGAGCCTAAACAAGCCGATG
GAP-43 RV	GGGCACTTTCCTTAGGTTTGGT
MAP1B FW	AGCCAGTCGAAGCCTACGTC
MAP1B RV	ATTCGCCCTCCCCTTCAGTG
F1_HuD-3'UTR-FW	TAATACGACTCACTATAGGGGCATTGAATGTTCTTTCATAGC
F1_HuD-3'UTR-RV	CTGTTTATAGCCCATCCTTC
F2_HuD-3'UTR-4-FW	TAATACGACTCACTATAGGGTGGGCTATAAACAGATGATCTT
F2_HuD-3'UTR-5-RV	CACAACAAACATGACAATATATCA
F3_HuD-3'UTR-6-FW	TAATACGACTCACTATAGGGGTTTGTGCTTTGTACGGTTA
F3_HuD-3'UTR-7-RV	CAATACTTTCCCTTGGAAATCATC

Supplementary Table 2. Sequences of the primers used for PCR or real-time qRT-PCR (indicated 5’ to 3’)

SUPPLEMENTARY REFERENCES

1. Brighi C, Salaris F, Soloperto A, Cordella F, Ghirga S, de Turris V *et al.* Novel fragile X syndrome 2D and 3D brain models based on human isogenic FMRP-KO iPSCs. *Cell Death and Disease* 2021; **12**:498.

2. Marrone L, Poser I, Casci I, Japtok J, Reinhardt P, Janosch A *et al.* Isogenic FUS-eGFP iPSC Reporter Lines Enable Quantification of FUS Stress Granule Pathology that Is Rescued by Drugs Inducing Autophagy. *Stem Cell Reports* 2018; **10**: 375–389.

3. Uhlén M, Fagerberg L, Hallström BM, Lindskog C, Oksvold P, Mardinoglu A *et al.* Proteomics. Tissue-based map of the human proteome. *Science* 2015; **347**: 1260419.

4. Agostini F, Zanzoni A, Klus P, Marchese D, Cirillo D, Tartaglia GG. catRAPID omics: a web server for large-scale prediction of protein-RNA interactions. *Bioinformatics* 2013; **29**: 2928–2930.
