# Supplementary material for: ALS-related FUS mutations alter axon growth in motoneurons and affect HuD/ELAVL4 and FMRP activity
Source: Commun Biol. 2021 Sep 1;4:1025. doi: 10.1038/s42003-021-02538-8 (PMC8410767; doi:10.1038/s42003-021-02538-8)
Supplement: Supplementary file 3 — Description of Additional Supplementary Files [file 42003_2021_2538_MOESM3_ESM.pdf]

Description of Additional Supplementary Files

File Name: Supplementary Data 1

Description: Predicted interactors of the HuD 3'UTR.

File Name: Supplementary Data 2

Description: Source data underlying graphs and charts presented in the figures.
