# Supplementary material for: ALS-related FUS mutations alter axon growth in motoneurons and affect HuD/ELAVL4 and FMRP activity
Source: Commun Biol. 2021 Sep 1;4:1025. doi: 10.1038/s42003-021-02538-8 (PMC8410767; doi:10.1038/s42003-021-02538-8)
Supplement: Supplementary file 6 — Reporting Summary [file 42003_2021_2538_MOESM6_ESM.pdf]

nature research

Corresponding author(s): Alessandro Rosa

Last updated by author(s): Jul 20, 2021

Reporting Summary

Nature Research wishes to improve the reproducibility of the work that we publish. This form provides structure for consistency and transparency in reporting. For further information on Nature Research policies, see our Editorial Policies and the Editorial Policy Checklist.

Statistics

For all statistical analyses, confirm that the following items are present in the figure legend, table legend, main text, or Methods section.

n/a	Confirmed	
☐	☒	The exact sample size (*n*) for each experimental group/condition, given as a discrete number and unit of measurement
☐	☒	A statement on whether measurements were taken from distinct samples or whether the same sample was measured repeatedly
☐	☒	The statistical test(s) used AND whether they are one- or two-sided<br>*Only common tests should be described solely by name; describe more complex techniques in the Methods section.*
☐	☒	A description of all covariates tested
☐	☒	A description of any assumptions or corrections, such as tests of normality and adjustment for multiple comparisons
☐	☒	A full description of the statistical parameters including central tendency (e.g. means) or other basic estimates (e.g. regression coefficient) AND variation (e.g. standard deviation) or associated estimates of uncertainty (e.g. confidence intervals)
☐	☒	For null hypothesis testing, the test statistic (e.g. F, t, r) with confidence intervals, effect sizes, degrees of freedom and P value noted<br>*Give P values as exact values whenever suitable.*
☒	☐	For Bayesian analysis, information on the choice of priors and Markov chain Monte Carlo settings
☒	☐	For hierarchical and complex designs, identification of the appropriate level for tests and full reporting of outcomes
☒	☐	Estimates of effect sizes (e.g. Cohen's *d*, Pearson's *r*), indicating how they were calculated

Our web collection on statistics for biologists contains articles on many of the points above.

Software and code

Policy information about availability of computer code

Data collection	No software was used
Data analysis	Prism - GraphPad version 8. catRAPID (http://s.tartaglialab.com/page/catrapid_group)

For manuscripts utilizing custom algorithms or software that are central to the research but not yet described in published literature, software must be made available to editors and reviewers. We strongly encourage code deposition in a community repository (e.g. GitHub). See the Nature Research guidelines for submitting code & software for further information.

Data

Policy information about availability of data

All manuscripts must include a data availability statement. This statement should provide the following information, where applicable:

- Accession codes, unique identifiers, or web links for publicly available datasets
- A list of figures that have associated raw data
- A description of any restrictions on data availability

All data generated or analysed during this study are included in this published article and its supplementary information files.

Field-specific reporting

Life sciences study design

All studies must disclose on these points even when the disclosure is negative.

Sample size	Sample size was not predetermined
Data exclusions	No data were excluded from the analyses
Replication	Experiments were replicated at least three time
Randomization	No randomization was perfomed
Blinding	No blinded experiments were conducted

Reporting for specific materials, systems and methods

We require information from authors about some types of materials, experimental systems and methods used in many studies. Here, indicate whether each material, system or method listed is relevant to your study. If you are not sure if a list item applies to your research, read the appropriate section before selecting a response.

Materials & experimental systems

n/a	Involved in the study	
☐	☒	Antibodies
☐	☒	Eukaryotic cell lines
☒	☐	Palaeontology and archaeology
☐	☒	Animals and other organisms
☒	☐	Human research participants
☒	☐	Clinical data
☒	☐	Dual use research of concern

Methods

n/a	Involved in the study	
☒	☐	ChIP-seq
☒	☐	Flow cytometry
☒	☐	MRI-based neuroimaging

Antibodies

Antibodies used	Anti-TUJ1 (T2200; Sigma-Aldrich), anti-GAP-43 (LS-C356053, Bio-techne), anti-MAP2 (ab5392, Abcam), anti-NEURITIN (AF283, R&D Systems), anti-mouse Alexa Fluor 488 (Thermo Fisher Scientific), anti-rabbit Alexa Fluor 594 (Immunological Sciences), anti-goat Alexa Fluor 594 (Immunological Sciences), anti-puromycin antibody (clone 12D10, MABE343; Merck), anti-HuD antibody (ab96474, Abcam), anti-FMRP (f4055; Sigma-Aldrich), anti-FMRP (ab17722; Abcam), rabbit monoclonal anti-human IgG (ab109489; Abcam), anti-GAPDH (MAB-10578; Immunological sciences), donkey anti-mouse IgG (H+L) (IS20404; Immunological Science), donkey anti-rabbit IgG (H+L) (IS20405; Immunological Science), anti-488-PHALLOIDIN (1:50; 49429, Sigma-Aldrich), Anti-TYR-TUBULIN clone YL1/2 (1:1000; MAB1864-I, Sigma-Aldrich), anti-Rat IgG (H+L), highly cross-adsorbed, CF™ 647 secondary antibody (1:500; SAB4600186, Sigma-Aldrich) produced in goat.
Validation	Antibodies were validated according to manufacturer instructions in human

Eukaryotic cell lines

Policy information about cell lines

Cell line source(s)	Human induced Pluripotent Stem cells (hiPSCs); HeLa cells
Authentication	FUS-WT and FUS-P525L-HM were previously published in Lenzi et al., 2015 (doi:10.1242/dmm.020099); HeLa cells were purchased from ATCC
Mycoplasma contamination	Lines were tested for mycoplasma contamination every three months
Commonly misidentified lines (See ICLAC register)	*Name any commonly misidentified cell lines used in the study and provide a rationale for their use.*

Animals and other organisms

Policy information about studies involving animals; ARRIVE guidelines recommended for reporting animal research

Laboratory animals	Mus musculus. The mouse strain used in this study (described in Devoy et al., 2017; 10.1093/brain/awx248) is designated B6N;B6J-Fustm1Emcf/H and commonly referred to as Fus Delta14. Primary MNs were isolated from E12.5-13.5 mouse embryos. Spinal cord samples were isolated from P81 mice

Wild animals	The study did not involve wild animals
Field-collected samples	The study did not involve samples collected from the field
Ethics oversight	All procedures for the care and treatment of animals were in accordance with the Animals (Scientific Procedures) Act 1986 Amendment Regulations 2012.

Note that full information on the approval of the study protocol must also be provided in the manuscript.
